# Quality Attributes and Health Risk Assessment of Sapodilla (*Manilkara zapota*) Subjected to Artificial Ripening Practices

**DOI:** 10.1002/fsn3.72565

**Published:** 2026-11-08

**Authors:** Usama Nasir, Amir Ismail, Muhammad Riaz, Asif Mahmood, Waheed Al Masry, Muhammad Latif, Farwa Aslam, Sher Ali, Carlos Augusto Fernandes de Oliveira, Muhammad Zahid Aslam

**Affiliations:** ^1^ Department of Food Science & Technology, Faculty of Food Science and Nutrition Bahauddin Zakariya University Multan Pakistan; ^2^ Department of Food Engineering, School of Animal Science and Food Engineering University of São Paulo Pirassununga São Paulo Brazil; ^3^ Department of Food Safety and Quality Management, Faculty of Food Science & Nutrition Bahauddin Zakariya University Multan Pakistan; ^4^ Department of Chemical Engineering, College of Engineering King Saud University Riyadh Kingdom of Saudi Arabia; ^5^ Department of Chemistry Rawalpindi Women University (RWU) Rawalpindi Punjab Pakistan; ^6^ Cotton Research Station Bahawalpur Pakistan

**Keywords:** artificial ripening, CaC_2_, ethylene, exposure, food safety, PTEs, risk, sapodilla

## Abstract

Artificial ripening of climacteric fruits remains a common postharvest practice in Pakistan and other developing countries, yet its effects on sapodilla ( *Manilkara zapota* ) quality and food safety remain uncharacterized. This study assessed the impact of regularly used artificial ripening methods, including ethylene gas, a commercial ethylene‐based ripener, and calcium carbide (CaC_2_), on physicochemical quality parameters, nutritional composition, selected antioxidant‐related indicators, and potentially toxic elements (PTEs) in sapodilla fruit pulp. Dietary exposure and non‐carcinogenic risks were evaluated using an established dietary intake model. CaC_2_ treatment significantly reduced total soluble solids, ascorbic acid, proximate nutritional composition parameters, and antioxidant indicators. In addition, CaC_2_‐treated fruits had levels of arsenic (0.22 mg/kg) and cadmium (0.14 mg/kg) exceeding their respective maximum levels internationally recommended. In contrast, controlled ethylene gas preserved key quality attributes of sapodilla with low PTE levels. The total hazard index (ΣTHI) for all treatments remained below the threshold (< 1.0), indicating no immediate non‐carcinogenic risk under the applied assumptions. However, carcinogenic risk estimates for arsenic (6.6 × 10^−4^) and cadmium (1.71 × 10^−3^) in CaC_2_‐treated fruits exceeded the conventional acceptable benchmark of 1 × 10^−4^, indicating a potential long‐term carcinogenic concern under the modeled exposure assumptions. These findings highlight the need for routine monitoring of ripening agents and enforcement of food control measures in Pakistan to meet regulatory demands and protect consumer health.

## Graphical abstract

Calcium carbide (CaC_2_) ripening severely compromises the quality and safety of sapodilla ( *Manilkara zapota* ) fruit. Compared with control and ethylene‐ripened fruit, CaC_2_ treatment reduced total soluble solids (TSS) and altered nutritional composition (protein, fat, fiber, vitamin C) and antioxidant potential (DPPH, phenolics, FRAP). It also increased potentially toxic elements (arsenic, lead, cadmium) above WHO limits. Ethylene‐based ripening retained better fruit quality and is a safer alternative for consumers.

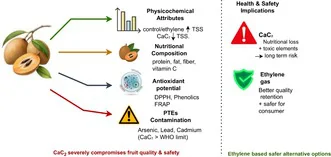

## 1 Introduction

Fruits constitute an essential component of human diets worldwide, providing carbohydrates, dietary fiber, vitamins, minerals, and other constituents relevant to nutritional adequacy (WHO 2025). In many low‐ and middle‐income countries, fruit production also represents a critical agricultural and economic sector. However, the expansion of fresh fruit markets has been accompanied by increasing concerns regarding postharvest handling practices, particularly artificial ripening, which may influence product quality, chemical safety, and consumer exposure to contaminants (Igiebor et al. 2023). These concerns are directly relevant to food control systems, where enforcement of safe ripening practices remains inconsistent.

Within Pakistan's horticultural sector, sapodilla ( *Manilkara zapota* ) is an economically relevant fruit that is widely marketed for fresh consumption. Although native to Central America, sapodilla is now cultivated across tropical and subtropical regions and is valued for its sweetness, texture, and contribution of dietary fiber, vitamin C, and minerals (Bangar et al. 2022; Gam et al. 2024). Major producing countries, including India, Mexico, and Brazil, integrate sapodilla into both domestic markets and regional trade (Miranda 2022; Singh et al. 2021). Despite this distribution, sapodilla has received limited attention in food safety and postharvest control research compared with higher‐volume fruits such as mango or banana, particularly regarding artificial ripening practices and associated contamination risks.

Fruit ripening is a natural physiological process that determines sensory attributes and market acceptability. In climacteric fruits, ethylene plays a central regulatory role in coordinating changes in texture, soluble solids, acidity, and color development (Singh et al. 2025; Tipu and Sherif 2024). In commercial supply chains, exogenous ripening agents are frequently applied to synchronize ripening, facilitate transport, and meet market demands (Hewajulige and Premaseela 2020). While controlled ethylene application is widely recognized, the continued use of unregulated or prohibited agents, particularly calcium carbide (CaC_2_) and certain commercial ripeners, remains a reported concern in several producing regions (Mondal et al. 2025; Nasir et al. 2024; Vidhya et al. 2025). Such practices have been associated with altered physicochemical characteristics, including changes in total soluble solids, acidity, and nutrient‐related parameters, which may compromise product quality and consistency (Islam et al. 2016; Maduwanthi and Marapana 2019; Nasir et al. 2025). In addition to quality attributes, artificial ripening raises food safety considerations related to chemical contamination. Several studies report reductions in phenolic content and antioxidant‐related indicators following artificial ripening, which are increasingly used as markers of fruit quality deterioration rather than direct predictors of health effects (Hussain et al. 2024; Marc et al. 2023; Sojinu et al. 2021). From a food control perspective, these changes may indicate inappropriate ripening conditions or chemical stress during postharvest handling.

More critically, ripening agents, environmental deposition, and agronomic inputs may introduce or mobilize potentially toxic elements (PTEs) in fruit matrices. Elements such as arsenic (As), cadmium (Cd), and lead (Pb) are non‐essential elements, being associated with adverse health effects even at low levels of exposure (Hossain et al. 2015; IARC 1993; Igiebor et al. 2023). CaC_2_ used for artificial ripening has been reported to contain impurities, including As and phosphide residues, which may contribute to elevated PTE concentrations in treated fruits (Ain et al. 2023; Okeke et al. 2022; Oladipupo et al. 2022). Although essential elements such as iron (Fe) and zinc (Zn) are nutritionally important, excessive intake through contaminated foods may also pose health concerns, underscoring the need for risk assessment (Jomova et al. 2025). While the risks associated with the use of CaC_2_ in fruits like bananas and mangoes have been established (Nasir et al. 2025, 2026), sapodilla remains under‐researched. Its unique biochemical properties, including high tannin levels and a short optimal ripening period, might influence its interaction with ripening agents. Furthermore, there is no available data on contamination by PTEs and dietary exposure to artificially ripened sapodilla from South Asian production systems. Therefore, the present study evaluated the effects of commonly applied artificial ripening practices on the physicochemical and nutritional quality indicators of sapodilla fruit, quantified associated PTE concentrations, and assessed potential consumer health risks using established dietary exposure metrics. By integrating quality parameters with contaminant monitoring and risk assessment, the study aimed to provide evidence relevant to food safety surveillance, regulatory consideration, and the development of safer postharvest ripening practices within sapodilla value chains.

## 2 Materials and Methods

### 2.1 Reagents, Standards and Equipment

Ethylene ripener sachets (unsaturated powder; reported purity > 90%; Ghani Global Group, Lahore, Pakistan) and commercial‐grade calcium carbide (CaC_2_; CAS 75‐20‐7) were purchased from a local supplier operating near the fruit market in Multan (Punjab, Pakistan). The ethylene ripener sachets represent a commonly used commercial product in Pakistan marketed for ethylene release. However, the manufacturer did not provide sufficient compositional specifications (e.g., active ingredients, release kinetics, impurities). Accordingly, sachet‐based ripening was evaluated as a real‐world practice rather than a dose‐controlled ethylene treatment. Ethylene gas for controlled exposure was generated using an Easy Ripe ethylene generator (Catalytic Generators, Norfolk, VA). Instrumentation included an ethylene gas analyzer (F‐950, Felix Instruments, Camas, WA), a digital pH meter (HI 99121, Hanna Instruments, Nușfalău, Romania), an Abbe refractometer (60/DR‐10–99, Bellingham + Stanley Ltd., Kent, UK), a double‐beam spectrophotometer (SPECORD 200 plus, Analytik Jena, Germany), and a flame atomic absorption spectrophotometer (240FS AA, Agilent, Santa Clara, CA). Hydrochloric acid (HCl), nitric acid (HNO_3_), perchloric acid (HClO_4_), and single−/multi‐element standards for potentially toxic elements (PTEs) were obtained from Merck (Darmstadt, Germany) and Sigma‐Aldrich (Saint Louis, MO). Unless otherwise stated, all reagents were analytical grade. All glassware and digestion vessels were acid‐washed and rinsed with deionized water before use to minimize background contamination.

### 2.2 Sample Collection and Ripening Treatments

Freshly harvested, unripe sapodilla ( *M. zapota* ) fruits (*n* = 120) with uniform size and apparent maturity were obtained from local farms near Multan, Pakistan, during the harvest period (December 2024–March 2025), and were randomly allocated into 12 experimental units, each consisting of 10 fruits. Before allocation to experimental units, these fruits were washed with double‐distilled water and air‐dried at room temperature (25°C–28°C; relative humidity 65%–75%) for approximately 30 min until surface moisture was visually absent. After that, the sapodilla fruit experimental units were subjected to four ripening treatments with three replications, as follows: Group 1 (control; natural ripening): Fruits were ripened under ambient conditions (25°C–30°C; 65%–75% relative humidity) until edible ripeness (4–5 days); Group 2 (CaC_2_ treatment): Fruits were placed in boxes containing two CaC_2_ packets (30 g each), wrapped in paper to minimize direct contact, and held under ambient conditions until edible ripeness (approximately 2 days); Group 3 (ethylene ripener sachets): Fruits were placed in boxes with two ethylene ripener sachets (3 g each), wrapped in paper and held under ambient conditions until edible ripeness (approximately 3–4 days). As the manufacturer does not specify the ethylene release rate or target concentration, this treatment reflects the prevalent market practice rather than a standardized ethylene dose; Group 4 (controlled ethylene gas): Fruits were exposed to 100 ppm ethylene gas in an airtight chamber for 48 h at 25°C, and then held under ambient conditions until edible ripeness (nearly 3 days).

To reduce initial maturity differences and provide maximum homogeneity of fruits before treatments, sapodillas free from any damage and showing a consistent peel color (light brown, firm) were chosen from the same harvest and matched for size (85–95 mm diameter). Treatments were executed simultaneously in ventilated rooms under similar conditions (25°C–30°C; 65%–75% relative humidity). Daily assessments of ripeness were made based on firmness and peel color (full brown) to ensure uniform maturity across groups. After completion of the ripening process and attainment of edible ripeness, the fruits in each unit were peeled, the pulps were collected separately, mixed, and homogenized to form one composite sample. Thus, three samples per treatment were obtained and analyzed for physicochemical properties, proximate parameters, antioxidant‐related indicators, and PTEs.

### 2.3 Physicochemical Analyses

Titratable acidity (TA) was determined according to Jalal et al. (2017), with standard titrimetric conditions. Briefly, 10 g of each sapodilla pulp subsample was diluted by adding 20 mL of distilled water, and 5 mL of the mixture was titrated with 0.1 N NaOH using phenolphthalein as the endpoint indicator. TA was expressed as % malic acid equivalent using Equation (1), and pH was measured using a calibrated digital pH meter (HI 99121, Hanna Instruments, Nușfalău, Romania).

(1) $$\mathrm{TA}\%=\frac{0.0067\times \text{volume of NaOH}\times 30}{5\times 10}\times 100$$

Total soluble solids (TSS) were determined using a refractometer (Abbe 60/DR‐10–99, Bellingham + Stanley Ltd., Kent, UK) by placing 1 mL of each diluted pulp subsample on the prism. Measurements were performed in duplicate for each diluted pulp subsample at room temperature and reported as °Brix. Sugar‐to‐acid ratio (TSS/TA) was calculated using matched TSS and TA values for each subsample. Ascorbic acid (vitamin C) was measured following the procedures as described by Javed et al. (2022). Ten millilter of pulp subsample were mixed with 100 mL of 0.4% oxalic acid, and 5 mL of the resulting mixture were titrated with 2,6‐dichlorophenolindophenol until a light pink endpoint. Results were reported as mg/100 mL.

### 2.4 Proximate Composition

Moisture, ash, crude protein, crude fat, crude fiber, and carbohydrate (%) were determined using AOAC methods (Latimer and Horwitz 2019). Minor procedural adjustments were limited to: (i) drying temperature/time for moisture determination, (ii) incineration temperature for ash, and (iii) solvent selection for fat extraction. All other procedural elements followed AOAC guidance. Any modifications were applied consistently across treatments to support comparability.

### 2.5 Total Phenolic Content

Total phenolic content (TPC) was measured using the Folin–Ciocalteu assay as described by Tabasum et al. (2016). Pulp subsample (20 g) was extracted twice with 80 mL of methanol: water (80: 20, *v/v*) and centrifuged at 9000 rpm for 15 min at 4°C. The extracts were combined and adjusted to a final volume of 60 mL. Next, 0.5 mL of the combined extract was mixed with 2.0 mL Folin–Ciocalteu reagent and 1.8 mL of 7.5% Na_2_CO_3_. After 60 min incubation, absorbance was measured at 765 nm using a double‐beam spectrophotometer (SPECORD 200 plus, Analytik Jena, Germany). TPC was reported as mg gallic acid equivalents (GAE)/g fresh weight (FW).

### 2.6 Antioxidant‐Related Indicators

Given the scope of this study, 2,2‐diphenyl‐1‐picrylhydrazyl (DPPH) radical‐scavenging activity and the ferric reducing/antioxidant power (FRAP) were treated as quality‐associated indicators that may reflect compositional changes during ripening treatments, rather than mechanistic endpoints. Extract preparation followed the method as described by Islam et al. (2023), using 0.1 mL of the extract obtained in Section 2.5 mixed with 2.9 mL of 0.1 mM DPPH solution. After incubation (30 min, dark, room temperature), absorbance was measured at 517 nm. Scavenging activity was calculated using Equation (2), where *A*_0_ is the control absorbance and *A*_S_ is absorbance in the presence of extract. DPPH results were reported as mg vitamin C equivalents/g FW, using the relevant calibration approach.

(2) $$\text{DPPH}\%=\left[\left(A_{0}-A_{S}\right)/A_{0}\right]\times 100$$

FRAP was determined according to Thaipong et al. (2006) and Neupane and Lamichhane (2020), with minor procedural modifications. FRAP reagent was prepared from acetate buffer, 2,4,6‐tris(2‐pyridyl)‐S‐triazine (TPTZ), and ferric chloride. The pre‐warmed reagent (4.0 mL; 37°C) was mixed with 0.1 mL of the extract obtained in Section 2.5 and incubated at 37°C. Absorbance was read at 593 nm against a blank after the stated incubation period, and FRAP results were reported as mg vitamin C equivalents/g FW.

### 2.7 Determination of Potentially Toxic Elements (PTEs)

PTE determination was restricted to pulp, rather than whole fruit, to provide a dietary exposure estimate more closely aligned with consumer intake under typical consumption practices, as peel is not consumed and washing procedures can reduce surface residues outside the fruits. Triplicate subsamples of composite pulps from each treatment were dried and ground into a fine powder. An aliquot (0.5 g) of each subsample was digested following Ubuoh et al. (2022), with minor modifications. In brief, samples were digested with 10 mL HNO_3_ and 5 mL HClO_4_ until complete digestion, then filtered and diluted to volume. Concentrations of As, Cd, Pb, Fe, and Zn were determined using a flame atomic absorption spectrophotometer (240FS AA, Agilent, Santa Clara, CA). Limits of detection (LOD) and quantification (LOQ) were established from 25 procedural blanks. LODs for As, Cd, Pb, Fe, and Zn were 0.0042, 0.0015, 0.0040, 0.05, and 0.2 mg/kg, respectively; LOQs were 0.0126, 0.0030, 0.010, 0.17, and 0.67 mg/kg, respectively. Samples exceeding defined limits (1%) or showing atypical repeatability were reanalyzed. Analytical accuracy was verified using a NIST Standard Reference Material (SRM 1570), and routine blank checks were included to control for laboratory contamination. Because CaC_2_ is a known potential source of inorganic impurities, strict contamination control (acid‐washed glassware, procedural blanks, SRM verification) was applied consistently across treatments to support comparisons.

### 2.8 Exposure Assessment and Risk Characterization

Dietary exposure and risk metrics were calculated for As, Cd, Pb, Fe, and Zn using standard intake models. Estimated daily intake (EDI) was calculated using Equation (3), where FIR is fruit ingestion rate, 𝐶 is the PTE concentration (mg/kg), and BW is the adult body weight (60 kg).

(3) EDI=FIR×C/BW

Non‐carcinogenic risk was determined according to the target hazard quotient (THQ) method by using Equation (4), where EF is exposure frequency (365 days/year), ED is exposure duration (67 years; life expectancy; Statista 2022), RfD is the oral reference dose, BW is the adult body weight (60 kg), and AT is the averaging time. Accordingly, the total hazard index (THI) was calculated as the sum of THQs across all assessed elements (THQ_PTE1_ + THQ_PTE2_ + … + THQ_PTEn_).

(4) $$\mathrm{THQ}=\left(\mathrm{EF}\times \mathrm{ED}\times \mathrm{FIR}\times C\right)/\left(\mathrm{RFD}\times \mathrm{BW}\times \mathrm{AT}\right)\times 10^{-3}$$

Cancer risk (CR) was calculated using Equation (5), where CSF is the cancer slope factor (USEPA 2015).

(5) CR=EDI×CSF

### 2.9 Quality Assurance and Method Validation

Analyses were conducted using validated protocols of AOAC (Latimer and Horwitz 2019). Instruments were calibrated using appropriate standards prior to analysis, and SRM‐based verification was applied for PTE analyses. Procedural blanks were included for digestion batches, and results outside predefined acceptance criteria (repeatability threshold: 1%) were reassessed.

### 2.10 Statistical Analysis

Results were reported as mean ± standard deviation of three samples per treatment group, with each sample containing the homogenized pulp from 10 fruits. Statistical analyses were performed using Statistix 8.1 (Softonic, Barcelona, Spain). After applying the Shapiro–Wilk test to the data for checking the normality, physicochemical, proximate composition, and PTE data were analyzed using one‐way ANOVA within a completely randomized design (CRD). Antioxidant‐related indicators were analyzed using a two‐way factorial ANOVA, with ripening treatment and extract type (methanol vs. aqueous) as factors. Treatment means were compared using the least significant difference (LSD) post hoc testing, considering *p* < 0.05.

## 3 Results and Discussion

### 3.1 Physicochemical and Compositional Analysis

Statistical analysis indicated significant treatment effects across measured parameters (*p* < 0.05), demonstrating that ripening approach influenced sapodilla quality attributes relevant to market acceptability and process control (Table 1). Naturally ripened fruit (control) showed a TSS value of 23.70 ± 0.75 °Brix, which was statistically comparable to fruit exposed to controlled ethylene gas (24.23 ± 0.45 °Brix). In contrast, the commercial ethylene ripener and CaC_2_ treatments resulted in significantly lower TSS values (19.40 ± 0.62 and 17.56 ± 0.40 °Brix, respectively), indicating reduced development of sweetness‐associated soluble solids under these practices (Table 1). TA ranged from 0.18% ± 0.01% malic acid equivalent in ethylene ripener‐treated fruit to 0.25% ± 0.01% malic acid equivalent in CaC_2_‐treated fruits. The TSS/TA ratio, commonly used as a maturity and sensory‐balance indicator, was highest in the control (125.03 ± 9.18) and ethylene gas‐treated fruit (121.30 ± 3.85), and lowest in CaC_2_‐treated fruits (70.36 ± 4.08), reflecting the least favorable sugar–acid balance under this treatment. pH also differed significantly among treatments, with the lowest values in CaC_2_‐treated fruits (4.63 ± 0.05) and the highest pH found in ethylene ripener‐treated fruits (5.20 ± 0.01). Vitamin C content was highest in the control (14.86 ± 0.35 mg/100 mL) and lowest in CaC_2_‐treated fruit (8.23 ± 0.25 mg/100 mL). Both ethylene gas and ethylene ripener treatments showed statistically similar intermediate values (13.20 ± 0.20 and 13.10 ± 0.20 mg/100 mL, respectively), which remained lower than the control as summarized in Table 1.

**TABLE 1:** Physicochemical quality parameters of sapodilla ( *Manilkara zapota* ) ripened using different artificial ripening agents.^a^

Treatment	TSS (°Brix)	TA (%)	TSS/TA	pH	Vitamin C (mg/100 mL)
Untreated (control)	23.70 ± 0.75a	0.19 ± 0.01bc	125.0 ± 9.18a	4.90 ± 0.01b	14.86 ± 0.35a
Ethylene gas treated	24.23 ± 0.45a	0.20 ± 0.01b	121.3 ± 3.85a	4.90 ± 0.01b	13.20 ± 0.20b
Ethylene ripener treated	19.40 ± 0.62b	0.18 ± 0.01c	107.9 ± 2.89b	5.20 ± 0.01a	13.10 ± 0.20b
CaC_2_ treated	17.56 ± 0.40c	0.25 ± 0.01a	70.36 ± 4.08c	4.63 ± 0.05c	8.23 ± 0.25c

*Note:* Values within the same column followed by different letters (a–c) differ significantly (*p* < 0.05).

Abbreviations: CaC_2_, calcium carbide; TA, titratable acidity; TSS, total soluble solids.

^a^ Data are reported as mean ± standard deviation of three composite samples per treatment, with each composite sample containing the homogenized pulp from 10 fruits.

In the present dataset, controlled ethylene gas exposure produced physicochemical values most closely aligned with natural ripening, whereas CaC_2_ treatment produced the largest deviations in TSS, TA, TSS/TA, pH, and vitamin C. These parameters are routinely used in postharvest quality control and, when interpreted alongside data on contaminants, can support risk‐based inspection priorities for ripening practices in informal supply chains. The two artificial ripening approaches differ operationally in ways relevant to food safety oversight. Ethylene gas (C_2_H_4_) is widely used under controlled postharvest conditions to replicate endogenous ethylene‐driven ripening responses (Tipu and Sherif 2024; Vidhya et al. 2017). In contrast, CaC_2_ generates acetylene (C_2_H_2_) upon hydrolysis (Equation 6), producing calcium hydroxide as a by‐product.

(6) $$\left(\mathrm{Ca}C_{2}+2H_{2}O\to C_{2}H_{2}+\mathrm{Ca}{\left(\mathrm{OH}\right)}_{2}\right)$$

Acetylene formation from CaC_2_ has been described in the literature (Schobert 2014). From a food control perspective, the practical concern is not acetylene itself but the impurity profile of technical‐grade CaC_2_. Industrial‐grade CaC_2_ is reported to contain As and phosphide‐bearing residues that may generate reactive byproducts (e.g., arsine, phosphine) upon hydrolysis (Ain et al. 2023; Nura et al. 2018; Okeke et al. 2022). These byproducts, alongside the strongly alkaline calcium hydroxide (Ca(OH)_2_) co‐product, may create a chemically stressful microenvironment at the fruit surface that could disrupt cell wall‐degrading enzyme activity, particularly amylase and invertase, which are responsible for starch‐to‐sugar conversion during ripening (Hewajulige and Premaseela 2020; Vidhya et al. 2025). The pronounced reductions in TSS observed here are therefore interpreted as quality degradation indicators consistent with impaired enzymatic ripening physiology, rather than as evidence of a specific disrupted biochemical pathway (Oladipupo et al. 2022). Consistent with this interpretation, the lower TSS values under ethylene ripener and CaC_2_ treatments (Table 1) contrast with typical increases expected during normal ripening (Bayoumi et al. 2023) and support prior observations that non‐standardized ripening practices can yield inconsistent compositional outcomes (Cissé et al. 2020). TSS values observed under controlled ethylene gas exposure are also consistent with prior reports using comparable ethylene concentrations and exposure durations in sapodilla (Tipu and Sherif 2024; Vidhya et al. 2017).

TA and pH differences further indicate treatment‐dependent shifts in acid‐related quality indices. Ethylene gas‐treated fruits maintained TA and pH values comparable to the control (Table 1), which is consistent with reports that controlled ethylene treatment can more closely track natural ripening patterns than non‐standardized chemical practices (Alonso‐Salinas et al. 2024; Vidhya et al. 2017). By contrast, CaC_2_‐treated fruit showed the highest TA and the lowest pH, yielding the lowest TSS/TA ratio, a profile associated with diminished sensory balance and reduced market quality (Cissé et al. 2020). However, these differences are presented as measurable quality deviations relevant to process control rather than as evidence of altered metabolic pathways.

Vitamin C decreased across artificial ripening treatments relative to the control, with the largest reduction observed under CaC_2_ treatment (Table 1). Declines in ascorbic acid during ripening have been reported in climacteric fruits and are commonly attributed to oxidative losses during maturation (Mditshwa et al. 2017). In this study, the markedly lower vitamin C in CaC_2_‐treated fruit (8.23 mg/100 mL vs. 14.86 mg/100 mL in the control) aligns with prior reports describing greater nutrient losses under CaC_2_‐based ripening compared with natural ripening (Hewajulige and Premaseela 2020; Lawaly 2022; Nasir et al. 2025; Nura et al. 2018). However, reactive oxygen species (ROS) generated under oxidative stress conditions induced by CaC_2_ impurities promote the irreversible oxidation of I‐ascorbic acid to dehydroascorbic acid and subsequently to 2,3‐diketogulonic acid (Enyioma‐Aloxie 2025; Mditshwa et al. 2017). Additionally, the alkaline Ca(OH)_2_ by‐product of CaC_2_ hydrolysis may destabilize ascorbic acid, which is known to degrade under non‐acidic conditions (Alberts et al. 2025). Together, these mechanisms support the interpretation of vitamin C reduction under CaC_2_ treatment as a consequence of both chemical oxidation and pH‐mediated instability, with direct implications for nutritional quality and consumer intake (Alberts et al. 2025; Enyioma‐Aloxie 2025). The smaller but significant reductions under ethylene gas and ethylene ripener treatments underscore that ripening protocols may require species‐specific optimization, as responses reported for other climacteric fruits are not necessarily transferable to sapodilla (Baidya et al. 2020; Bayoumi et al. 2023).

### 3.2 Compositional Quality Indicators (Proximate Composition)

Proximate composition also differed significantly among ripening treatments (*p* < 0.05), as shown in Table 2. CaC_2_‐treated sapodilla exhibited the highest moisture (78.10% ± 0.20%) and ash content (0.53% ± 0.005%), while the control showed the highest fat (0.86% ± 0.02%), protein (0.68% ± 0.01%), and fiber (6.94% ± 0.10%). Ethylene gas and ethylene ripener treatment fruits generally had small but significant reductions in fat, protein, and fiber relative to the control, whereas CaC_2_‐treated fruit showed the lowest values for fat (0.66% ± 0.01%), protein (0.55% ± 0.01%), and fiber (5.20% ± 0.10%). Carbohydrate content was highest in the control and ethylene ripener‐treated fruit (19.01% ± 0.30% and 19.06% ± 0.20%, respectively), and lowest under CaC_2_ treatment (17.64% ± 0.15%).

**TABLE 2:** Proximate composition of sapodilla ( *Manilkara zapota* ) ripened using different artificial ripening agents.^a^

Treatment	Moisture (%)	Ash (%)	Fat (%)	Protein (%)	Fiber (%)	Carbohydrate (%)
Untreated (control)	76.16 ± 0.35b	0.49 ± 0.01b	0.86 ± 0.02a	0.68 ± 0.01a	6.94 ± 0.10a	19.01 ± 0.30a
Ethylene gas treated	74.96 ± 0.15c	0.50 ± 0.01b	0.80 ± 0.01b	0.66 ± 0.01b	6.52 ± 0.10b	18.90 ± 0.15a
Ethylene ripener treated	75.06 ± 0.30c	0.50 ± 0.01b	0.85 ± 0.03a	0.68 ± 0.01ab	6.52 ± 0.10b	19.06 ± 0.20a
CaC_2_ treated	78.10 ± 0.20a	0.53 ± 0.01a	0.66 ± 0.01c	0.55 ± 0.01c	5.20 ± 0.10c	17.64 ± 0.15b

*Note:* Values within the same column followed by different letters (a–c) differ significantly (*p* < 0.05).

Abbreviation: CaC_2_, calcium carbide.

^a^ Data are reported as mean ± standard deviation of three composite samples per treatment, with each composite sample containing the homogenized pulp from 10 fruits.

The increased ash content in CaC_2_‐treated fruit should be treated as a screening‐level quality indicator rather than a nutritional benefit, particularly because industrial‐grade CaC_2_ is reported to contain mineral impurities (including arsenic‐ and phosphorus‐containing residues) that may contribute to chemical contamination concerns (Abubakar et al. 2020; Hussain et al. 2024; Nura et al. 2018). Because moisture responses to CaC_2_ have been reported inconsistently across fruits and study conditions (Adeyemi et al. 2018; Nuhu et al. 2020; Olubiyo et al. 2022), the present moisture increase is reported conservatively as a treatment‐associated outcome without extrapolating the mechanism. Variability across studies is plausibly influenced by cultivar differences, ripening environment (temperature/humidity), and CaC_2_ application details (dose and exposure duration), which has practical relevance for enforcement. Non‐standardized market practices can produce heterogeneous quality and safety profiles, complicating compliance assurance (Bamiwola et al. 2024; Cissé et al. 2020; Deshi et al. 2024; Hussain et al. 2024).

The reductions in protein and fiber under CaC_2_ treatment (Table 2) are most plausibly attributable to two interacting processes. First, proteolytic enzyme activity (protease/peptidase) is known to accelerate during chemically induced or stress‐accelerated ripening in climacteric fruits, resulting in degradation of structural and storage proteins (Lawaly 2022; Vidhya et al. 2025). Second, the decline in dietary fiber under CaC_2_ treatment may reflect accelerated cell‐wall polysaccharide degradation, specifically pectin solubilization and hemicellulose hydrolysis driven by elevated polygalacturonase and cellulase activities that accompany faster, chemically induced ripening (Baidya et al. 2020; Bayoumi et al. 2023; Hewajulige and Premaseela 2020; Yüksel et al. 2025). Taken together, the physicochemical (Table 1) and proximate composition (Table 2) data indicate that controlled ethylene gas treatment most closely approximated natural ripening quality indices, while CaC_2_ treatment produced the largest quality deviations. Given the known regulatory scrutiny of CaC_2_ use and impurity concerns reported in the literature, these findings support prioritizing CaC_2_‐ripened fruit for targeted surveillance and compliance checks, particularly when coupled with contaminant measurements and risk metrics presented in subsequent sections (Nasir et al. 2025; Oladipupo et al. 2022).

### 3.3 Total Phenolic Content and Antioxidant‐Related Quality Indicators

TPC and antioxidant‐related assay outcomes (DPPH and FRAP values) differed significantly among ripening treatments (*p* < 0.05), indicating treatment‐dependent changes in compositional quality indices, as shown in Figures 1 and 2. In this study, TPC, DPPH, and FRAP are interpreted as quality‐associated indicators that reflect retention or loss of phenolic‐associated reducing capacity during ripening practices, rather than as mechanistic endpoints or direct predictors of consumer health benefit. Across both methanol (MeOH) and aqueous extracts, naturally ripened sapodillas (controls) consistently showed the highest TPC (Figure 1). Both ethylene gas and ethylene ripener treatments had lower TPC than controls, while CaC_2_‐treated fruit exhibited the lowest TPC, indicating the greatest reduction in phenolic‐associated constituents under this treatment (Figure 1). This pattern is consistent with reports indicating that artificial ripening, mainly CaC_2_‐based ripening, can be associated with greater reductions in phenolic content relative to natural ripening (Tassoult et al. 2021). From a food control perspective, reduced TPC under market‐based ripening practices is relevant as an indicator of diminished compositional integrity, which may co‐occur with other quality deviations that warrant postharvest practice scrutiny.

**FIGURE 1:**
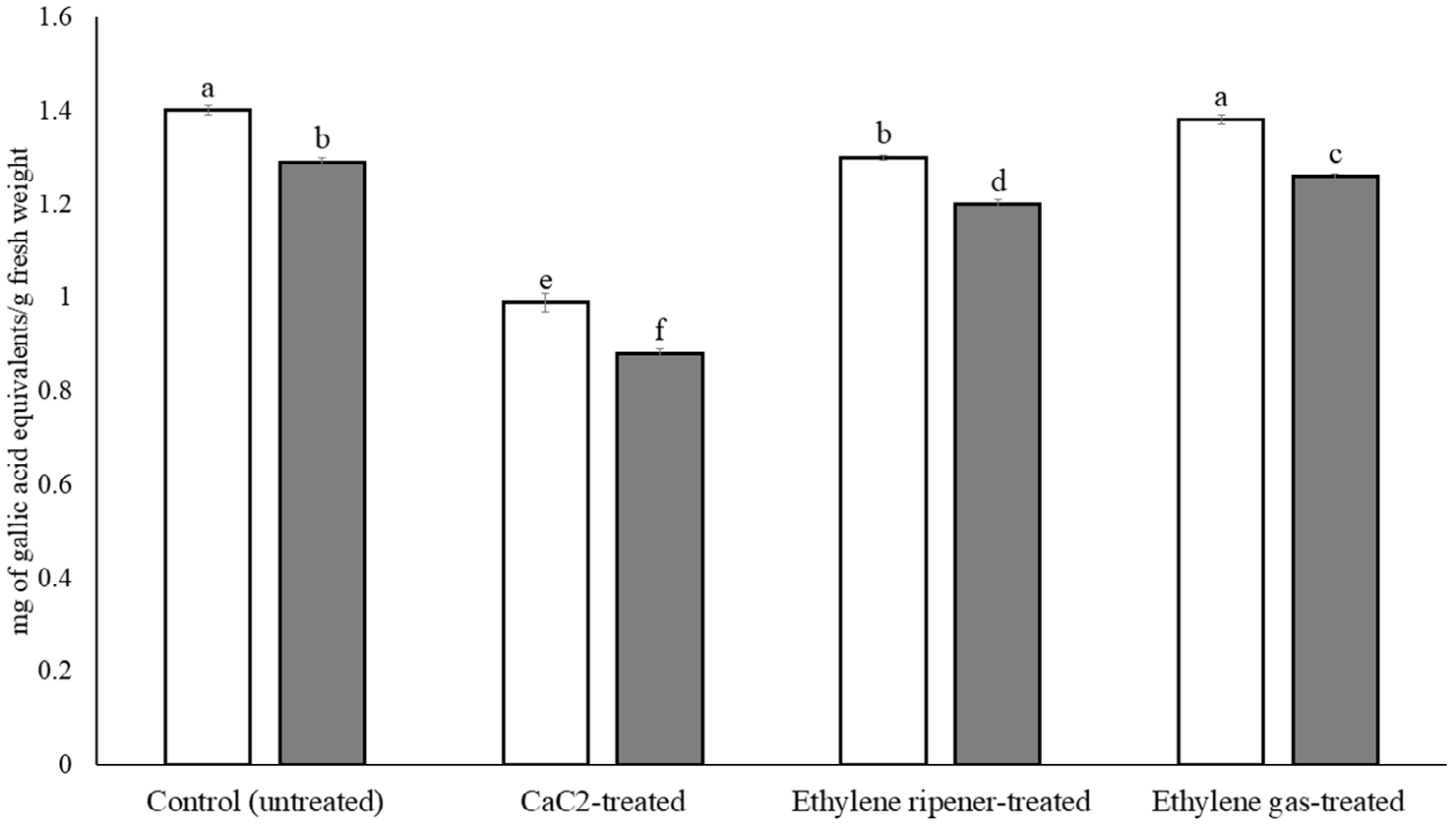
Total phenolic content in sapodilla ( *Manilkara zapota* ) samples ripened with different artificial ripening agents. 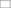 Methanolic extract; 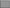 Aqueous extract. Results are expressed as mean ± standard deviation from three replicates. ^a–f^Bars with different letters differ significantly *(p* < 0.0001) by factorial two‐way ANOVA with LSD test.

**FIGURE 2:**
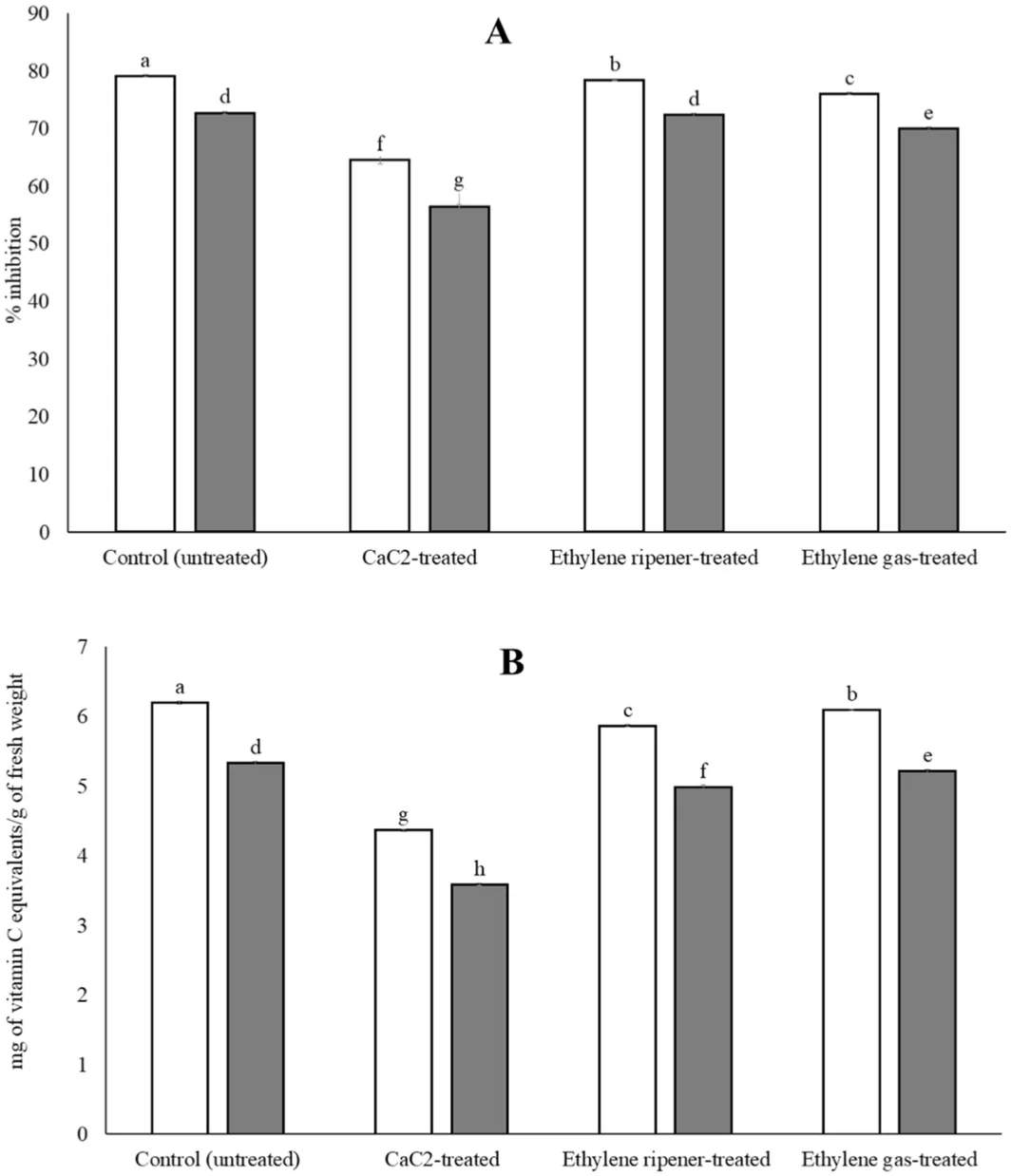
Antioxidant activity in sapodilla ( *Manilkara zapota* ) samples ripened with different artificial ripening agents, assessed by two methods: (A) Free radical scavenging activity (DPPH); (B) Ferric reducing antioxidant power (FRAP). 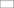 Methanolic extract; 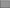 Aqueous extract. Results are expressed as mean ± standard deviation from three replicates. (a–h) Bars with different letters differ significantly *(p* < 0.0001) by factorial two‐way ANOVA with LSD test.

DPPH % inhibition and FRAP followed the same treatment ordering observed for TPC, with the control showing the highest values and CaC_2_‐treated fruit the lowest (Figure 2). Specifically, FRAP values ranged from 4.37 to 6.20 and 3.59 to 5.33 mg VCE/g FW in methanol and water extracts, respectively, with control fruits consistently exhibiting the highest reducing power and CaC_2_‐treated fruits the lowest (Figure 2). DPPH activity showed a comparable gradient: methanol extracts of control fruits demonstrated the highest activity (79.13%), whereas CaC_2_‐treated fruits showed the lowest activity (64.62%). Water extracts showed slightly lower DPPH activity across all treatments, which is consistent with solvent‐dependent extraction efficiency for phenolic‐associated constituents. Across all treatments and extraction solvents, DPPH activity was strongly and positively correlated with FRAP (*r* = 0.9713), supporting internal consistency between these two assay outputs within the dataset. These findings align with earlier reports for sapodilla showing higher FRAP and DPPH values in naturally ripened fruit and lower values under chemical ripening conditions (Alrashood et al. 2020; Tamsir et al. 2020; Woo et al. 2013). Similar trends have also been reported for other fruits, where ripening was associated with reductions in phenolic‐associated antioxidant indices, and non‐standardized ripening practices highlighted these declines (Lawaly 2022; Tassoult et al. 2021; Vidhya et al. 2025).

The observed reductions in TPC, DPPH, and FRAP under CaC_2_ treatment are consistent with two physiological mechanisms commonly reported in the artificial ripening literature. First, polyphenol oxidase (PPO) activity, which catalyzes the oxidation of phenolic compounds to quinones, is known to be upregulated under conditions of cellular stress and accelerated ripening. CaC_2_‐induced chemical stress may therefore accelerate phenolic degradation through PPO‐mediated oxidation, reducing the measurable phenolic pool (Li et al. 2023; Tassoult et al. 2021; Wojdyło et al. 2013). Second, the ROS environment associated with CaC_2_ impurities (discussed in Section 3.1) can directly oxidize and deplete low‐molecular‐weight phenolic compounds, which are among the most redox‐reactive species in fruit tissue (Fasanya et al. 2019; Lawaly 2022). Generally, the findings shown in Figures 1 and 2 indicate that natural ripening preserved the highest phenolic‐associated quality indices, ethylene‐based treatments provided intermediate reductions, and CaC_2_ treatment resulted in the lowest values. For regulatory and monitoring contexts, these results support the use of compositional quality indicators (TPC, DPPH, FRAP) as screening tools to characterize the quality consequences of market ripening practices and to inform inspection priorities, particularly where CaC_2_ use is suspected or documented, while remaining anchored to the measured data and the study's contaminant and risk assessment outcomes (Lawaly 2022; Tassoult et al. 2021; Vidhya et al. 2025).

### 3.4 Analysis of Potentially Toxic Elements

The concentrations of five PTEs (As, Pb, Cd, Fe and Zn) quantified in sapodilla pulp subsamples following different ripening treatments are presented in Table 3. Statistically significant differences among treatments were observed for all measured elements (*p* < 0.05), indicating that ripening method influenced the elemental profile of the fruit edible portion. Sapodilla ripened with CaC_2_ exhibited the highest mean concentrations of all assessed PTEs. When evaluated against the reference limits cited from FAO/WHO (2011), As in CaC_2_‐treated fruits (0.22 ± 0.01 mg/kg) exceeded the reported permissible limit of 0.1 mg/kg, while cadmium (0.14 ± 0.01 mg/kg) exceeded the limit of 0.05 mg/kg. Pb concentration in CaC_2_‐treated fruits (0.26 ± 0.04 mg/kg) remained below the cited limit of 0.3 mg/kg, while iron and zinc concentrations were also within their respective reference limits. In contrast, As was below the limit of detection in ethylene gas‐treated samples and control, and PTE concentrations in ethylene gas‐ and ethylene ripener‐treated fruits were generally lower than those observed under CaC_2_ treatment and within the cited FAO/WHO limits. Across treatments, the relative abundance of elements followed the order Fe > Zn > Pb > As > Cd.

**TABLE 3:** Concentrations (mg/kg of fresh weight) of potentially toxic elements (PTEs) in sapodilla ( *Manilkara zapota* ) pulp ripened using different ripening methods.^a^

Treatment	As	Pb	Cd	Fe	Zn
Untreated (control)	< LOD	0.01 ± 0.001b	< LOD	12.80 ± 0.02b	8.23 ± 0.02c
CaC_2_ treated	0.22 ± 0.02a	0.26 ± 0.04a	0.14 ± 0.01a	17.96 ± 0.01a	10.13 ± 0.02a
Ethylene gas treated	< LOD	0.01 ± 0.002b	0.002 ± 0.001c	13.26 ± 0.01b	8.56 ± 0.01b
Ethylene ripener treated	0.05 ± 0.01b	0.01 ± 0.002b	0.03 ± 0.02b	13.00 ± 0.02b	8.46 ± 0.01bc
FAO/WHO (2011) reference limits	0.10	0.30	0.05	NR	50

*Note:* Values within the same column followed by different letters (a–c) differ significantly (*p* < 0.05).

Abbreviations: As, arsenic; CaC_2_, calcium carbide; Cd, cadmium; Fe, iron; LOD, limit of detection; NR, not reported; Pb, lead; Zn, zinc.

^a^ Data are reported as mean ± standard deviation of three composite samples per treatment, with each composite sample containing the homogenized pulp from 10 fruits.

These results indicate that CaC_2_‐based ripening is associated with a less favorable contaminant profile in sapodilla pulp. While the study does not establish the source of contamination, elevated As and Cd levels in CaC_2_‐treated fruit are consistent with previous reports noting that technical‐grade CaC_2_ may contain inorganic impurities, including As‐bearing compounds (Abubakar et al. 2020; Ikhajiagbe et al. 2022; Nura et al. 2018). The presence of such contaminants is of particular concern because As and Cd are non‐essential elements associated with adverse health effects at low levels of chronic exposure (IARC 1993; Nasir et al. 2025; Okeke et al. 2022). In this study, the commercial formulation of ethylene ripener provided lower PTE concentrations in pulp subsamples than CaC_2_‐treated fruits (Table 3), while ethylene gas‐treated fruits exhibited a contaminant profile comparable to the control. Similar patterns of elevated PTE concentrations in artificially ripened fruits, particularly those treated with CaC_2_, have been reported in other studies (Deshi et al. 2024; Lakade et al. 2019; Lawaly 2022; Nasir et al. 2025). Conversely, some investigations demonstrated lower or variable PTE levels in artificially ripened fruits (Hussain et al. 2024; Nuhu et al. 2020), highlighting that environmental conditions, differences in analytical methods and ripening practices may influence PTE concentrations in the evaluated fruits. Within this context, the present findings reinforce the need for routine monitoring and quality control of ripening agents, particularly where CaC_2_ use persists outside formal regulatory limits. The PTE results demonstrate that ripening practice is a determinant of contaminant burden in sapodilla pulp and support prioritizing CaC_2_‐ripened fruit for targeted inspection and compliance testing to ensure consumer safety.

### 3.5 Exposure Assessment and Health Risk Characterization

Potential dietary exposure and associated health risks from consuming sapodilla subjected to different ripening treatments were evaluated using EDI, THQ, ΣTHI and lifetime CR. Based on the exposure assumptions applied in this study, EDI values for all assessed PTEs were below the provisional tolerable daily intake (PTDI) limits referenced from FAO/WHO (2011) across all treatments, as shown in Table 4. Compared with the controls, higher EDI values were generally observed for fruit subjected to artificial ripening, with the largest increases associated with CaC_2_ treatment. In particular, Pb intake from CaC_2_‐ripened sapodilla (5.2 × 10^−4^ mg/kg/day) exceeded the control EDI but remained substantially below the PTDI value. Similar patterns were observed for As and Cd, where CaC_2_‐treated fruits provided higher EDI values than other treatments but did not exceed PTDI thresholds under the modeled intake scenario.

**TABLE 4:** Estimated daily intake (EDI, expressed as mg/kg body weight/day)^a^ of potentially toxic elements (PTEs) from consumption of sapodilla ( *Manilkara zapota* ) ripened using different ripening agents.

Treatment	As	Pb	Cd	Fe	Zn
Control	0	2.0 × 10^−5^	0	2.56 × 10^−2^	1.65 × 10^−2^
CaC_2_ treated	4.4 × 10^−4^	5.2 × 10^−4^	2.8 × 10^−4^	3.59 × 10^−2^	2.03 × 10^−2^
Ethylene gas treated	0	2.0 × 10^−5^	4.0 × 10^−6^	2.65 × 10^−2^	1.71 × 10^−2^
Ethylene ripener treated	1.0 × 10^−4^	2.0 × 10^−5^	6.0 × 10^−5^	2.60 × 10^−2^	1.69 × 10^−2^
FAO/WHO (2011) PTDI reference	1.0 × 10^−2^	3.0 × 10^−3^	2.5 × 10^−2^	2.0 × 10^−1^	3.0 × 10^0^

Abbreviations: As, arsenic; bw, body weight; CaC_2_, calcium carbide; Cd, cadmium; Fe, iron; Pb, lead; PTDI, provisional tolerable daily intake; Zn, zinc.

^a^ Calculated as EDI = (FIR × C)/adult body weight (60 kg), where FIR is the assumed daily intake of sapodilla, and C is the mean PTE concentration (mg/kg). Concentrations below the analytical limit of detection were treated as zero for exposure estimation.

Table 5 presents the THQ and ΣTHI values calculated for assessing the non‐carcinogenic risks of all PTEs evaluated, and the CR values obtained for As, Pb and Cd in the sapodilla pulps. For all treatments, individual THQ and ΣTHI values remained below the conventional threshold of 1.0, indicating a low non‐carcinogenic risk under the assumptions adopted in the study. The highest ΣTHI was observed for CaC_2_‐ripened sapodilla, with As and Cd contributing the largest shares, reflecting their elevated concentrations in this treatment (Table 3). Conversely, carcinogenic risk estimates had greater differentiation among treatments. For most PTEs and treatments, CR values fell within or below the acceptable range of 10^−6^ to 10^−4^ (USEPA 2015). However, the CR associated with Cd exposure from CaC_2_‐ripened sapodilla (1.71 × 10^−3^) exceeded the upper benchmark of 1 × 10^−4^, while the As‐associated CR for the same treatment (6.6 × 10^−4^) also exceeded this benchmark (Table 5). Pb‐associated CR values remained comparatively low across treatments. These results indicate that, within the applied risk assessment framework, CaC_2_‐ripened sapodilla represents the highest‐priority scenario for potential long‐term carcinogenic risk, driven primarily by As and Cd exposure.

**TABLE 5:** Target hazard quotient (THQ), total hazard index (ΣTHI), and cancer risk (CR) associated with the consumption of sapodilla ( *Manilkara zapota* ) ripened using different artificial ripening agents.

Treatment	THQ (×10^−3^)^a^					ΣTHI (×10^−3^)^b^	Cancer risk (CR)^c^		
	As	Pb	Cd	Fe	Zn		As	Pb	Cd
Control	0	0.006	0	0.037	0.055	0.103	0	1.7 × 10^−7^	0
CaC_2_ treated	1.468	0.149	0.280	0.051	0.068	2.016	6.6 × 10^−4^	4.42 × 10^−6^	1.71 × 10^−3^
Ethylene gas treated	0	0.006	0.004	0.038	0.057	0.105	0	1.7 × 10^−7^	2.44 × 10^−5^
Ethylene ripener treated	0.334	0.006	0.060	0.037	0.056	0.493	1.5 × 10^−4^	1.7 × 10^−7^	3.66 × 10^−4^

Abbreviations: As, arsenic; CaC_2_, calcium carbide; Cd, cadmium; Fe, iron; Pb, lead; Zn, zinc.

^a^ THQ values > 1.0 indicate a potential non‐carcinogenic health risk.

^b^ ΣTHI represents the sum of individual THQs for As, Cd, Pb, Fe, and Zn.

^c^ Acceptable lifetime cancer risk benchmark is 1.0 × 10^−4^ (USEPA 2015).

These results underline the importance of integrating contaminant monitoring with exposure and risk assessment to inform regulatory decision‐making of fruit ripening practices. Although average EDI values did not exceed PTDI limits, elevated CR values for specific PTEs under CaC_2_ treatment highlight that compliance with intake thresholds alone may not fully capture long‐term risk considerations. Accordingly, the combined evidence from PTE concentrations (Table 3) and risk metrics (Tables 4 and 5) supports stricter control of CaC_2_ use, enhanced surveillance of artificially ripened fruits, and promotion of safer, regulated ripening alternatives to minimize consumer exposure to hazardous elements.

## 4 Limitations of This Study

Several limitations should be considered when interpreting the findings of this study. First, the study was conducted in a single geographic region (Multan, Punjab, Pakistan), which limits the generalizability of results to other agroclimatic zones, cultivars, or production seasons, where soil composition, agronomic inputs, and post‐harvest handling may differ. Additionally, the commercial ethylene ripener used lacked specific compositional data, making its evaluation a reflection of real‐world market practices rather than a controlled intervention, complicating comparisons with controlled ethylene gas treatments. While the quantification of PTEs in this study was conducted in pulp, which aligns with consumer intake, surface residues and peel‐associated contamination were not assessed. Therefore, future studies should cover multiple regions, different cultivars, and consumer subgroups, as well as the use of dose‐controlled ripening protocols and evaluation of peel‐associated PTEs to strengthen the evidence for regulatory consideration.

## 5 Conclusion

Results of this study indicate that the type of ripening technique is a significant determinant of physicochemical quality, nutritional composition, antioxidant‐related indicators, and chemical safety profile of sapodilla. Controlled ethylene gas treatment most closely reproduced the natural ripening effect across all measured parameters, also generating the lowest PTE concentrations in the edible pulp. CaC_2_ treatment produced the largest quality deviations, including reduced TSS, ascorbic acid, protein, fiber, and phenolic‐associated indicators alongside As and Cd levels exceeding FAO/WHO reference limits. Although the total hazard index (ΣTHI) remained below 1.0 for all treatments, CR estimates for As and Cd under CaC_2_ treatment exceeded the conventional benchmark of 1 × 10^−4^, indicating a non‐negligible long‐term exposure concern under the modeled scenario. These findings stress the need for stringent regulatory surveillance of CaC_2_‐ripened sapodilla in Pakistan. Future work should extend the evaluation to multiple regions, cultivars, and seasons, incorporate dose‐controlled ripening protocols, and assess consumer subgroup risks to further inform regulatory decision‐making and postharvest governance in sapodilla supply chains.

## Author Contributions

**Usama Nasir:** conceptualization, methodology, validation, investigation, writing – original draft. **Amir Ismail:** conceptualization, investigation, funding acquisition, methodology, validation, writing – review and editing, project administration, supervision. **Muhammad Riaz:** conceptualization, methodology, validation, investigation, writing – review and editing, supervision, project administration, funding acquisition. **Asif Mahmood:** formal analysis, data curation. **Waheed Al Masry:** validation, writing – review and editing. **Muhammad Latif:** data curation, formal analysis. **Farwa Aslam:** methodology, investigation, writing – review and editing. **Sher Ali:** validation, writing – review and editing. **Carlos Augusto Fernandes de Oliveira:** conceptualization, investigation, funding acquisition, methodology, writing – review and editing, project administration, supervision, validation. **Muhammad Zahid Aslam:** validation, writing – review and editing.

## Funding

This work was supported by Fundação de Amparo à Pesquisa do Estado de São Paulo (2022/03952‐1).

## Conflicts of Interest

The authors declare no conflicts of interest.

## Data Availability

The data that support the findings of this study are available from the corresponding author upon reasonable request.
